# Genomic instability as a major mechanism for acquired resistance to EGFR tyrosine kinase inhibitors in cancer

**DOI:** 10.1007/s13238-021-00855-6

**Published:** 2021-07-28

**Authors:** Bing Liu, Daniela Duenas, Li Zheng, Karen Reckamp, Binghui Shen

**Affiliations:** 1 Department of Cancer Genetics and Epigenetics, Beckman Research Institute of City of Hope, 1500 East Duarte Road, Duarte, CA 91010 USA; 2 Division of Medical Oncology, Cedars-Sinai Medical Center, 8700 Beverly Blvd, West Hollywood, CA 90048 USA

The mutation-mediated overexpression of epidermal growth factor receptor tyrosine kinase (EGFR TK) and its activation play an important role in the cellular proliferation and epithelial tumorigenesis. A series of inhibitors targeting the intracellular tyrosine kinase (TK) domain of EGFR have been developed and applied to clinical practice. Although these inhibitors safely and effectively restrain tumor cell proliferation and prolong survival in some patients, acquired resistance ultimately arises. DNA mutations contribute to drug-induced cancer-cell resistance. Genomic instability, especially DNA replication and repair error, provides the major source for DNA mutations. Identifying the central mechanisms underlying the generation and selection of resistance mutations may provide critical opportunities for novel regimens in combating drug resistance. In this review, we provide an overview of EGFR tyrosine kinase inhibitors (TKIs) in non-small cell lung carcinoma (NSCLC) treatment and their challenges. We also discuss the major source of genomic instability in TKI resistance and hypothesize that the maintenance of DNA replication and repair machinery might be used to develop novel treatment regimens for patients with NSCLC.

EGFR is a member of the receptor tyrosine kinase (RTK) family (Lemmon et al., 2014). The activation of cytosol membrane EGFR via binding EGF-like ligands initiates receptor dimerization, phosphorylation of its own tyrosine residues, and activation of downstream signaling pathways. Aberrant EGFR activation, due to its single-nucleotide substitutions in exons 18–21, in-frame duplications/insertions in exon 20, or short in-frame deletions in exon 19, can amplify a series of downstream pro-oncogenic signaling pathways including JAK/STAT, PI3K/AKT/mTOR, PLC/PKC/NFκB and MEK/ERK. These pathways aim to support and benefit cancer cell survival, proliferation and tissue differentiation (El-Hashim et al., 2017) (Fig. 1). Three generations of EGFR TKIs have been developed to specifically target EGFR mutations to the kinase domain in NSCLC. However, an ever-increasing number of mutation-mediated resistances are inevitable (Zhang, 2016b).

**Figure 1:**
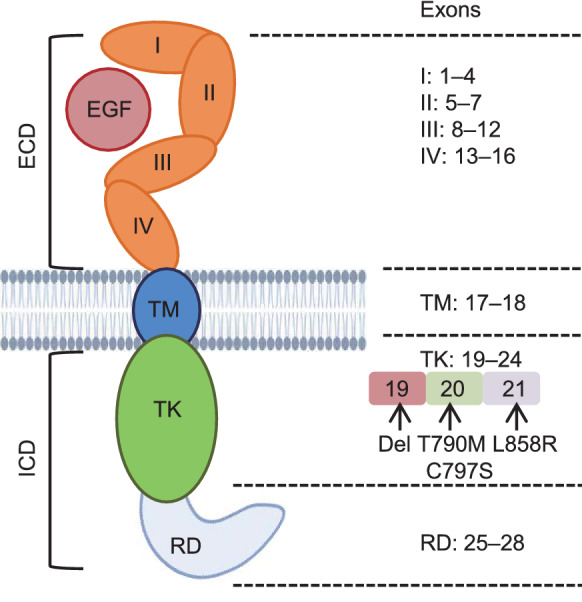
The EGFR protein structure and corresponding gene exons. Exons 1–16 encode extracellular domains I-IV (orange) which can form the ligand interaction conformation. Exons 17–18 encode the transmembrane domain (blue) for connecting extracellular domains and intracellular domains. Exons 19–24 encode tyrosine kinase domain. Exon 19 deletion and exon 21 L858R mutation are original mutations that cause constant activation of tyrosine kinase activity in non-small cell lung carcinoma. Exon 20 T790M is the dominant secondary mutation acquired in response to the 1st and 2nd generation TKIs, while exon 20 C797s mutation is the secondary mutation acquired in response to the 3rd generation TKI osimertinib. Exon 25–28 encode C-terminal phosphorylation domain which mediates the interactions between the receptor and downstream substrates upon receptor activation. Abbreviations: EGF, epidermal growth factor; ECD, extracellular domain; TM, transmembrane; ICD, intracellular domain; TK, tyrosine kinase; RD, regulatory/phosphorylation domain

Due to the mutation-mediated destabilization of the EGFR TK domain, abnormal activation of EGFR constitutively propagates EGFR TK activity and downstream pro-oncogenic signaling pathways to drive cancer cell survival and proliferation. Moreover, the expressions and interactions of a vast amount of genes and proteins are significantly changed during EGFR activation, suggesting the profound and extensive role of EGFRs involvement in the diverse signaling networks of cells (Waters et al., 2012). Although there are various pharmaceutical agents that target the proteins involved in the EGFR-mediated network of NSCLC, EGFR TKI remain the first line of treatment (Dong et al., 2021). Activated EGFR can promote protein nuclear translocation or redistribute to the nucleus via autophosphorylation, where it functions in DNA replication and repair which is an important process for genome fidelity (Wang et al., 2010, 2012). Reduced EGFR TK activity in response to TKIs might impair DNA replication and repair processes and boost the production of mutations for cancer progression. Both pre-existing and de novo generation of genome wide mutations have been observed *in vivo* and *in vitro*, suggesting the EGFR TKIs initiate genomic instability to generate and select for mutations that confer resistance to their inhibition of cancer cell growth and induction of apoptosis (Hata et al., 2016). Therefore, a better understanding of how TKIs initiate genomic instability is critical for developing novel strategies to control NSCLC progression.

Three generations of EGFR TKIs, as the ATP mimetic inhibitors, have been developed so far to target the most common somatic *EGFR* mutations, including the exon 19 deletion and the L858 mutation in exon 21, which account for more than 85% of all EGFR mutations (Molina-Vila et al., 2009) (Table 1). The first-generation TKIs, including gefitinib and erlotinib, reversibly bind to the active site of the EGFR TK domain to prevent ATP binding for kinase activity transduction. Acquired resistance to first-generation TKIs most often arises due to the T790M mutation, occurring in around 50% of patients (Ma et al., 2011). Despite difficulties in the detection of pre-existing mutations, pre-treatment sample evaluations have been reported to harbor T790M mutations (Watanabe et al., 2015). Furthermore, using a T790M-negative *in vitro* system, it was also demonstrated that EGFR TKI treatment can generate T790M mutations de novo (Kim et al., 2012). Specifically, a study conducted by Kadi and colleagues, found that NFκB activation by TKIs promotes activation-induced cytidine deaminase (AICDA) expression, which leads to the deamination of 5-methylcytosine to thymine and finally generates the T790M mutation (El Kadi et al., 2018). Second-generation TKIs, including afatinib and dacomitinib, irreversibly bind to the mutated and wild-type EGFR, as well as the receptors from bypass signaling pathways, such as HER2 (Genova et al., 2014; Baraibar et al., 2020), to provide a more sustained and potent EGFR inhibitory function. However, the acquired mutations including T790M still occur over the course of treatment. Recently, the mutant-selective third generation TKI osimertinib was designed to selectively and covalently bind to the C797 residue of EGFR at the ATP-binding pocket edge of its TK domain to repress EGFR-activating mutations while sparing wild-type receptors (Greig, 2016). Previously, osimertinib was used as second-line treatment in NSCLC patients who developed T790M-mediated resistance to first- and second-generation TKIs (Zhang, 2016a). Recent studies suggested that it was more effective to use osimertinib as first-line therapy (Aguilar-Serra et al., 2019). However, the most common mutation, C797S in exon 20, has been observed in around 10–26% of patients with resistance to second-line osimertinib treatment and around 7% of patients with resistance to first-line treatment (Mehlman et al., 2019).

**Table 1:** Summary of key EGFR-TK inhibitors

	EGFR-TKIs	Trade name	Primary target	Mechanism of action	Dominant secondary mutation	Clinical trial number	Refs
First generation	Gefitinib	Iressa	EGFR	Reversible	T790M	NCT02959749	Muhsin et al. (2003)
	Erlotinib	Tarceva	EGFR	Reversible	T790M	NCT00364351	Bareschino et al. (2007)
	Lapatinib	Tyverb	EGFR; ErbB2	Reversible	T790M	NCT01125566	Moy et al. (2007)
	Icotinib	Conmana	EGFR	Reversible	T790M	NCT03231501	Shi et al. (2013)
Second generation	Afatinib	Gilotrif	EGFR; ErbB2; ErbB4	Irreversible	T790M	NCT02094573	Dungo and Keating (2013)
	Dacomitinib	Vizimpro	EGFR; ErbB2; ErbB4	Irreversible	T790M	NCT01000025	Wu et al. (2017)
	Neratinib	Nerlynx	EGFR; ErbB2; ErbB4	Irreversible	T790M	NCT01000025	Sequist (2010)
Third generation	Osimertinib	Tagrisso	EGFR T790M	Irreversible	C797S	NCT01449461	Greig (2016)

In addition to EGFR-dependent mutations, an array of alternative EGFR-independent bypass signaling pathways may be concurrently activated to exacerbate tumor heterogeneity and therapeutic difficulty under EGFR-TKI treatment. Thus, the combination strategies which target both oncogenic mutations of EGFR and EGFR-independent bypass signaling pathways have been applied to delay the acquisition of resistance to some extent in many cases. The most common mechanism for bypass signaling-mediated acquired resistance, in 5%–50% of patients receiving second-line osimertinib treatment and 7%–15% of patients receiving first-line osimertinib treatment, occurs due to high levels of *MET* gene amplification (Ou et al., 2016). The MET gene amplification can induce constitutive activation of the EGFR downstream pro-oncogenic signaling pathways, such as JAK/STAT, PI3K/AKT/mTOR, PLC/PKC/NFκB and MEK/ERK pathways (Rotow et al., 2020; Yu et al., 2021). Thus, MET inhibitors have been used in combination with osimertinib to overcome acquired resistance (Awad et al., 2019). Another common bypass alteration is the overexpression of Anexelekto (AXL), a tyrosine kinase receptor, which can interact with EGFR and has been reported to be associated with poor osimertinib responses (Taniguchi et al., 2019). The combination of AXL inhibitor cabozantinib with osimertinib is a promising strategy to prolong osimertinib sensitivity (Reckamp et al., 2019). However, clinical trials are needed to confirm the long-term response for these strategies in patients. Beyond these EGFR-dependent and independent alterations to chromosomal DNA, there are yet other routes promoting drug resistance at the genomic level. For example, extrachromosomal DNA (ecDNA), which can be unevenly segregated into daughter cells due to the lack of a centromere, has been found in nearly half of human cancers (Turner et al., 2017). It has been reported that mutant EGFR ecDNA is eliminated in tumor cells during TKI treatment, inducing drug resistance, but restores after drug withdrawal (Nathanson et al., 2014).

How do spontaneous somatic mutations originate, particularly under the stresses such as drug administration? A series of repair processes coordinate with DNA replication to reduce spontaneous mutagenesis and maintain DNA fidelity. The key determinant of DNA fidelity depends on DNA polymerases selectivity and proof reading functions, which are important in organized incorporation of nucleotides into DNA during replication (Ludmann and Marx, 2016; Bębenek and Ziuzia-Graczyk, 2018; Xing et al., 2019). Moreover, during the DNA synthesis in lagging strand, the DNA polymerases, such as Polα and primase, de novo synthesize the RNA primer and α-segment with high error rates. Elimination of those errors relies on the structure-specific nucleases, such as FEN1 and DNA2, which are involved in the accurate RNA primer removal and the editing of α-segment errors. Deficiency in these processes will not only leave in those errors, but also generate duplication mutations due to failure of RNA primer removal which then exacerbates the mutation burden (Zheng and Shen, 2011; Li et al., 2018; Zheng et al., 2020). The mismatch repair (MMR) signaling is another determinant of DNA replication fidelity by correcting the remaining mismatches after DNA replication to promise DNA fidelity under homeostasis (Haradhvala et al., 2018). However, EGFR TKIs might hijack the key DNA replication/repair components and impair these processes for promoting single tumor cells to acquire multi-level molecular alterations at the genetic, transcriptional, post-translational, and epigenetic levels and ultimately boost intrinsic tumor heterogeneity for genome wide mutation generation (Majem and Remon, 2013).

It is established now that EGFR TK possesses more than 200 substrates (https://www.phosphosite.org/homeAction.action; https://string-db.org/network/9606.ENSP00000275493). These protein substrates are not only components of oncogenic signaling pathways (JAK/STAT, PI3K/AKT/mTOR, PLC/PKC/NFκB and MEK/ERK) that promote cancer cell survival and proliferation, but are also involved in DNA replication machinery (Fig. 2). Although EGFR inhibition with TKIs may suppress pro-oncogenic pathways, it may also result in other unintended effects such as the impairment of DNA replication fidelity and promotion of somatic mutagenesis. Supporting evidence is available for such a hypothesis. Cao and colleagues have recently demonstrated that the expression of heat shock protein 70 (HSP70), an ATP-dependent molecular chaperone, is reduced by EGFR TKI treatment (Cao et al., 2018). They found that HSP70 physically interacts with multiple enzymes in base excision repair (BER) and DNA replication pathways. Thus, the down regulation of HSP70 in response to TKIs enhances the gene mutation rate and attenuates BER to facilitate acquired resistance. Activated EGFR from the cytosol membrane can redistribute to the nucleus via the Golgi and endoplasmic reticulum (ER) under the assistance of translocon (Wang and Hung, 2009). Nuclear EGFR plays an essential role in stabilization of DNA replication and repair proteins, such as proliferation cell nuclear antigen (PCNA). PCNA recruits and coordinates DNA synthesis machinery to ensure accurate DNA replication and repair at the replication forks (Moldovan et al., 2007). Nuclear EGFR mediates the phosphorylation of PCNA in its chromatin-bound form, which is important for maintenance of PCNA stability and protection of chromatin-bound PCNA from proteasome-dependent degradation via lysine polyubiquitination (Wang et al., 2006; Lo et al., 2012). Blockage of its phosphorylation by EGFR TKIs may impair the assembly of the replication and repair machinery and lead to genome instability. The other important example is DNA-dependent protein kinase (DNA-PK) which is required for rejoining double-strand breaks to repair DNA. The nuclear EGFR can physically interact with DNA-PK and trigger DNA-PK phosphorylation (Bandyopadhyay et al. 1998; Dittmann et al., 2005a). Impaired DNA-PK phosphorylation due to the blockage of EGFR nuclear translocation reduces DNA-PK activity and promotes DNA damage (Dittmann et al., 2005b, 2008). These pieces of evidence suggest that EGFR TKIs may not cause DNA damage directly but can impair DNA replication and repair machinery due to degradation of the component proteins that missing phosphorylation protection and then lead to the acquired genome wide mutations.

**Figure 2:**
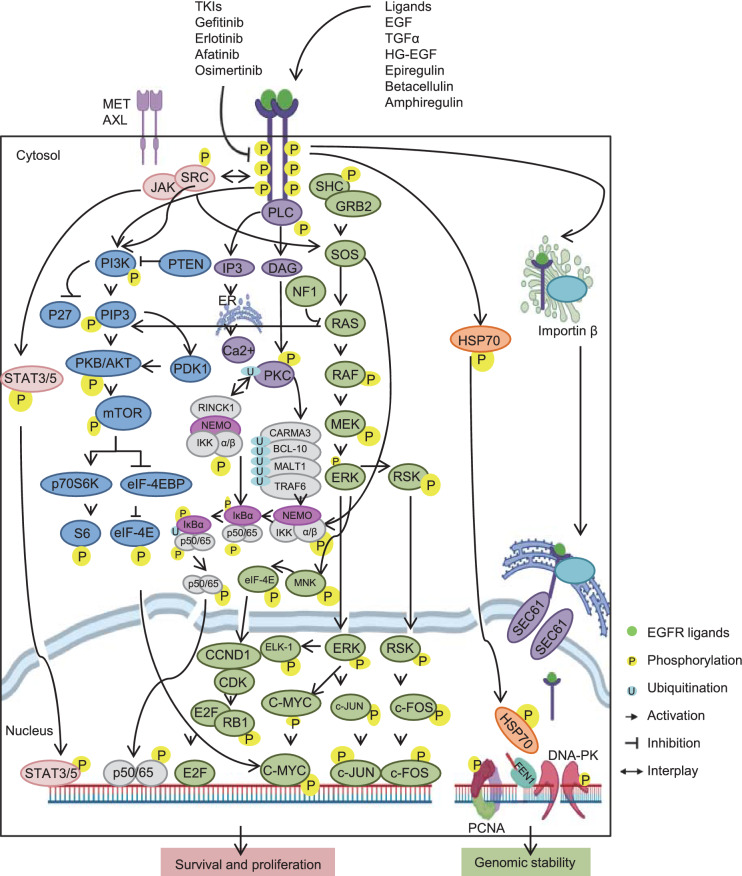
The network of EGFR-dependent phosphorylation cascade. This network is created based on KEGG database and current literatures. The EGFR ligands, such as EGF, TGFα, HG-EGF, Epiregulin, Betacellulin, and Amphiregulin, interact with EGFR extracellular domain to activate it via inducing its TK domain trans-autophosphorylation. The other kinases, such as SRC, are also able to phosphorylate EGFR from cytosol and be phosphorylated by activated EGFR in reverse. The expression of a number of genes is significantly changed during EGFR activation, which is accompanied with the various dynamic modifications, particularly phosphorylation. The most characterized pro-oncogenic signaling pathways phosphorylated and activated upon EGFR activation are listed at the left, including JAK/STAT, PI3K/AKT/mTOR, PLC/PKC/NFκB and MEK/ERK signaling pathways. The downstream transcription factors, including STAT3/5, p50/p65 NFκB dimer, E2F, c-MYC and c-JUN/c-FOS, play the oncogenic function to benefit cancer cell survival and proliferation. Furthermore, the stabilities of DNA replication and repair proteins, which are controlled by EGFR activation and nuclear EGFR, are illustrated at the right, including HSP70, PCNA and DNA-PK. Inhibition of EGFR TK activity with TKIs not only blocks pro-oncogenic pathways, but also DNA replication and repair pathways which are important for maintaining genomic stability. Genomic instability is the major source for resistance mutation generation, which might reduce EGFR TKI efficiency and activate the receptors from bypass signaling pathways, such as MET and AXL receptors, to further support cancer progression. Thus, maintaining genomic stability, especially by protecting the expression and stability of DNA replication and repair components, may forestall the generation and evolution of tumor cell mutations, ultimately reducing drug resistance. Abbreviations: SRC, Proto-oncogene tyrosine-protein kinase Src; JAK, Janus kinase; STAT3/5, Signal transducer and activator of transcription3/5; PI3K, Phosphatidylinositol-4,5-bisphosphate 3-kinase; PTEN, Phosphatase and tensin homolog; PIP3, Phosphatidylinositol (3,4,5)-trisphosphate; PDK, 3-phosphoinositide-dependent protein kinase; PKB, Protein kinase B; AKT, v-Akt murine thymoma viral oncogene homolog; mTOR, Mechanistic target of rapamycin kinase; p70S6K, Ribosomal protein S6 kinase; eIF-4EBP, Eukaryotic translation initiation factor 4E binding protein; EIF4E, Eukaryotic translation initiation factor 4E; S6, Ribosomal protein S6; PLC, Phospholipase C; IP3, Inositol trisphosphate; DAG, Diacylglycerol; PKC, Protein kinase C; RINCK1, E3 ligase RING finger protein that interacts with C kinase 1; NEMO, Inhibitor of nuclear factor kappa B kinase regulatory subunit gamma; IKK, inhibitor of nuclear factor kappa B kinase subunit; CARMA3, Caspase recruitment domain family member 10; BCL-10, B cell lymphoma protein 10; MALT1, Mucosa-associated lymphoid tissue lymphoma translocation gene 1; TRAF6, TNF receptor associated factor 6; p50, NFκB Subunit 1; p65, RELA proto-oncogene, NFκB subunit; SHC, SHC adaptor protein 1; GRB2, Growth factor receptor bound protein 2; SOS, Ras/Rac guanine nucleotide exchange factor; RAS, Rat sarcoma virus; RAF, Rapidly accelerated fibrosarcoma; MEK, Mitogen-activated protein kinase kinase; ERK, Extracellular signal-regulated kinase; RSK, MAP kinase-activated protein kinase; MNK, ATPase copper transporting alpha; CCND1, Cyclin D1; CDK, Cyclin dependent kinase; E2F, E2F transcription factor; RB1, RB transcriptional corepressor 1; ELK-1, ETS transcription factor; c-MYC, Myc proto-oncogene protein; c-JUN, Transcription factor AP-1; c-FOS, AP-1 transcription factor subunit; HSP70, Heat shock 70 kDa protein; SEC61, Translocon subunit alpha 1; PCNA, Proliferation cell nuclear antigen; DNA-PK, DNA-dependent protein kinase; FEN1, Flap endonuclease 1; ER, Endoplasmic reticulum

Mimicking the therapeutic approach of the HIV “cocktail” regimens, the recently approved combination therapeutic regimen with the 3^rd^ generation EGFR TKI osimertinib and MET inhibitor Tepotinib is based on the observation that MET gene amplification bypasses the EGFR TK activity and upregulates the EGFR downstream pro-oncogenic signaling pathway in EGFR TKI-treated patients (Markham, 2020). The combinations of EGFR-TKIs with immune checkpoint inhibitors are an emerging trend in NSCLC treatment (Jin et al., 2020). The immune checkpoint inhibitors include the ones for the programmed cell death-1 receptor and its ligand (PD-1/PD-L1) and cytotoxic T-lymphocyte-associated antigen 4 (CTLA-4) (Johnson et al., 2014). However, these regimens do not include a strategy to minimize the mutations from its origin. Our proposal for a therapeutic avenue is to protect the integrity of the DNA replication machinery and to suppress the S-phase cell cycle checkpoint activation in order to avoid drug-induced mutations. To maintain DNA replication fidelity, the employment of a proteasome inhibitor in combination with EGFR-TKI therapies may preserve DNA replication protein stability and should be considered. As previously mentioned, HSP70 is susceptible to proteasome degradation in response to EGFR TKIs. Recent experiments in our laboratory showed that when administering a protease inhibitor, Bortezomib, there is a clear reduction of HSP70 degradation. Furthermore, after conducting cellular based assays we observed a significant improvement to EGFR TKI sensitivity as well. A vast majority of the somatic mutations are generated through error prone DNA synthesis including aberrant Okazaki fragment maturation in the S-phase cells, which requires extended S-phase time and activation of the checkpoints. Combining the checkpoint inhibitors, such as ATR inhibitors, with TKIs, to reduce the acquired resistance is an alternative proposed strategy (Vendetti et al., 2015). In summary, EGFR serves a multifaceted role in cells and its inhibition can prove deleterious effects to genome stability. As we continue to face the persistent challenge of drug resistance in EGFR TKIs, we turn our focus to maintaining the integrity of DNA replication and repair pathways. By conserving the fidelity of the DNA replication and repair machinery we may increase drug sensitivity and impede tumor cell mutations that aid in the acquisition of drug resistance.

## Abbreviations

AICDA: activation-induced cytidine deaminase
AXL: anexelekto
BER: base excision repair
CTLA-4: cytotoxic T-lymphocyte-associated antigen 4
DNA-PK: DNA-dependent protein kinase
ecDNA: extrachromosomal DNA
EGFR: epidermal growth factor receptor
ER: endoplasmic reticulum
HSP70: heat shock protein 70
MMR: mismatch repair
NSCLC: non-small cell lung carcinoma
RTK: receptor tyrosine kinase
TK: tyrosine kinase
TKIs: tyrosine kinase inhibitors
PD-1/PD-L1: programmed cell death-1 receptor and its ligand
PCNA: proliferating cell nuclear antigen
